# Research progress on influencing factors and intervention methods of protein-energy wasting in maintenance hemodialysis patients: A literature review

**DOI:** 10.1097/MD.0000000000041592

**Published:** 2025-02-21

**Authors:** Jiquan Zhang, Fan Xu, Wei Qing, Peimin Zhuang, Xingying Xu

**Affiliations:** a Nephrology Department, Deyang People’s Hospital, Deyang, Sichuan Province, China; b Oncology Department, Deyang People’s Hospital, Deyang, Sichuan Province, China; c Department of Nephrology, The First Hospital of Putian City, Putian, China.

**Keywords:** energy wasting, influencing factors, intervention, literature review, maintenance hemodialysis, malnutrition, protein

## Abstract

Protein-energy wasting (PEW) has high incidence in hemodialysis patients and refers to a state of decreased protein and energy substance. With the extensive development of hemodialysis in patients with end-stage kidney disease, most patients with maintenance hemodialysis have the problem of PEW, which is one of the common complications of maintenance hemodialysis patients, resulting in decreased quality of life and increased mortality. This article searches and analyzes the literature in recent years to summarize the current influencing factors and common interventions of PEW in maintenance hemodialysis patients, which will provide reference for subsequent nutritional management of maintenance hemodialysis patients.

## 1. Introduction

Chronic kidney disease (CKD) affects more than 850 million people globally, with the prevalence rate increasing year after year, and the prevention and treatment of CKD has become a global public health problem.^[1,2]^ The Global Burden of Disease Study predicts that CKD will be the fifth leading cause of death in the world by 2040.^[3]^ With the continuous decline of kidney function, CKD will progress to end-stage kidney disease, kidney replacement therapy is the main treatment for kidney disease. Due to the shortage of kidney resources, high risk and cost of kidney transplantation, postoperative complications and posttransplantation rejection, maintenance hemodialysis (MHD) has become the mainstream choice for maintaining kidney function in patients with end-stage kidney disease.^[4]^ The total number of people on maintenance dialysis in China has reached 749,000, and is on the rise year by year, with the overall mortality rate of hemodialysis patients at 14.4%.^[5]^ Protein-energy wasting (PEW) is a common complication in patients with CKD and is strongly associated with poor patient prognosis. The concept of PEW was first proposed in 2008 by the International Society of Renal Nutrition and Metabolism (ISRNM), it refers to a state of “malnutrition” in which various nutritional and metabolic abnormalities occur in patients with CKD, resulting in a decrease in the body’s protein energy reserves.^[6]^ The clinical presentation of PEW is usually a syndrome of abnormal serum biochemistry, weight loss, body fat loss, and progressive skeletal muscle wasting. Although long-term hemodialysis prolongs the survival of patients, as the course of treatment progresses, patients develop reduced intake, anorexia, nutrient deficiencies, and a rise in tissue catabolism caused by the activation of inflammatory cytokines,^[7,8]^ resulting in the development of PEW in patients with MHD. According to statistics, the PEW prevalence among dialysis studies was 28% to 54%,^[9]^ and showed an increasing trend, which caused widespread concern among healthcare professionals. PEW is associated with decreased physical function, increased risk of falls, reduced quality of life, and increased mortality in MHD patients,^[10,11]^ 15% to 20% of MHD patients die of malnutrition.^[12]^ PEW not only leads to poor clinical outcomes, but also increases the financial burden of patients.^[13]^ Decreased nutritional intake and hypermetabolic mechanisms are both important factors in PEW. Hemodialysis purifies metabolic wastes produced by food intake, but dietary intake is usually controlled to limit potassium and phosphate and to avoid excess body fluids, which usually leads to malnutrition in patients with MHD. Currently, there is a notable deficiency in comprehensive discussions regarding the influencing factors and intervention methods related to PEW among MHD patients in China. This study aims to summarize and analyze the latest research on the influencing factors and intervention strategies for PEW in MHD patients by conducting a thorough literature review. The findings are intended to provide guidance for preventing malnutrition and implementing effective health education initiatives for MHD patients.

## 2. PEW diagnostic criteria

ISRNM states that the diagnosis of PEW can be made from the following 4 aspects, with at least 1 of each indicator meeting the criteria, and at least ≥ 3 of the 4 indicator systems to diagnose PEW.^[6]^ The details are shown in Table 1.

**Table 1:** PEW diagnostic criteria.

Indicator	Diagnostic criteria
Serum chemistry	Serum prealbumin <30 mg/dL (for MHD patients only)
	Serum albumin <3.8 g/dL
	Serum cholesterol <1 g/L
Body mass	Body mass index <23 kg/m^2^
	Unintentional weight loss of >5% within 3 mo or >10% within 6 mo
	Total body fat percentage <10%
Muscle mass	Loss of muscle mass (>5% within 3 mo, or >10% within 6 mo)
	Reduced mid-arm muscle circumference area (reduction >10%% in relation to 50th percentile of reference population)
Dietary intake	Unintentional protein intake in dialysis patients <0.8 g/(kg/d) for at least 2 mo
	Unintentional energy intake <25 kcal/(kg/d) for at least 2 mo

1 kcal = 4.184 kJ.

PEW = protein-energy wasting.

## 3. Influencing factors of PEW in MHD patients

### 3.1. Social and demographic factors that influence PEW

Age and education level are the influencing factors of MHD patients’ PEW. For example, the older the age, the worse the nutritional status of MHD patients.

#### 3.1.1. Age

Tayyem^[14]^ recruited 178 patients with MHD and 56.2% were moderately malnourished and age was inversely related to the nutritional status of the patients. A multicenter clinical observational study in Europe showed that the incidence of PEW was higher in elderly dialysis-treated patients aged 65 years and above, and the incidence also increased with age.^[15]^ This may be due to the fact that as age increases, elderly patients are more prone to PEW due to the gradual deterioration of body functions, a significant decrease in mobility, a lack of or even inability to carry out functional exercises, and more pronounced muscle atrophy in various body indicators.

#### 3.1.2. Education level

Rezeq^[16]^ found that education level has a significant impact on nutritional status. MHD patients without education have a greater risk of malnutrition, and MHD patients with college education have a higher nutritional score, indicating better nutritional status. This may be due to the fact that patients with higher education have a higher grasp of health education knowledge, are aware of the harm caused by malnutrition risk, and can reasonably master the intake methods of energy and protein to meet their nutritional needs. Health care workers need to provide educational guidance to MHD patients to improve the nutritional status and health status of MHD patients.^[17]^ Depending on the patient’s level of education, health education should adopt a combination of written materials, videos and other educational means to stimulate the learning interest of MHD patients and help them master nutrition-related knowledge. In addition, a diet list was developed for MHD patients based on their dietary preferences and nutrient intake guidelines. According to the nutritional knowledge of patients and various biochemical indicators, the diet of patients was adjusted to improve dietary compliance and nutritional status.

### 3.2. Insufficiency of intake

The main cause of PEW is insufficient nutrient intake, especially protein and energy (calories).^[18]^ On the one hand, the accumulation of toxic substances in the patient’s body leads to a decrease in appetite, which affects about 35% to 60% of end-stage MHD patients with reduced nutrient intake,^[19]^ and on the other hand, altered protein anabolic–catabolic metabolism is a key factor in malnutrition in MHD patients.^[20,21]^ Jimenez et al^[22]^ pointed out that gastrointestinal symptoms are common in MHD patients with protein and energy (calorie) deficiencies, which causes malnutrition risk in MHD patients. Dawson^[18]^ analyzed 298 MHD patients and 38% of them had changes in taste sensation and that nausea, vomiting, anorexia and dry mouth were upper gastrointestinal symptoms were significantly correlated with malnutrition. Further loss of amino acids and other nutrients during dialysis makes it more challenging to improve the nutritional status of MHD patients.^[23]^

### 3.3. Inflammatory conditions

Potential sources or triggers of systemic inflammation in patients with MHD typically include the following: biocompatibility of dialysis membranes, accumulation of uremic toxins, inadequate dialysis, infections during dialysis, and chronic diseases (diabetes, cardiovascular disease, etc) Kaizu^[24]^ pointed out that muscle consumption in MHD patients is closely related to inflammatory index, especially the serum interleukin-6 level, and inhibiting these inflammatory responses in patients can improve malnutrition and restore muscle mass. Inflammatory cytokines such as tumor necrosis factor-α (TNF-α) and interleukin-1 stimulate proteolytic metabolism and inhibit muscle protein synthesis through activation of the ubiquitin–proteasome system and the calpain pathway, leading to loss of muscle mass and muscle atrophy. Inflammatory cytokines are able to promote cellular autophagy, leading to the degradation of intracellular proteins and lipids and affecting cellular energy production and metabolic homeostasis. In addition, they are also able to inhibit the signaling of growth factors such as insulin-like growth factor-1, further suppressing anabolic metabolism. Ikizler^[25]^ also pointed out that systemic inflammation is an important factor affecting the nutritional status of MHD patients. Stenvinkel^[26]^ suggested that interactions between inflammatory cytokines and other factors (acidosis, anemia, and hormonal disorders) may contribute to PEW in maintenance hemodialysis patients, which explains the association of PEW with the synthesis of catabolic or antimetabolic inflammatory cytokines. Inflammatory cytokines TNF-α and interleukin-6 can induce insulin resistance, which not only affects glycemic control, but may also affect the synthesis and metabolism of thyroid hormones, leading to hypothyroidism or hyperthyroidism. Additionally, they may also affect energy metabolism and lead to disorders of lipid metabolism, such as increased body energy expenditure, as well as enhancing the production of very low density lipoproteins and reducing lipoprotein lipase activity. Acidosis affects the functioning of the gastrointestinal tract, leading to reduced digestion and absorption, which affects the appetite of MHD patients, leading to nausea and vomiting, thus reducing nutrient intake. Acidosis leads to a decrease in intracellular pH, which affects the activity of a variety of enzymes, including those involved in protein synthesis, and can result in decreased protein synthesis. Acidosis also leads to increased energy expenditure by the body, as cells require additional energy to maintain acid–base balance, and this increased energy demand promotes the breakdown of proteins and fats.

Malnutrition in MHD patients is multifactorial, and the malnutrition caused by acidosis may occur independently of inflammation or be exacerbated by an inflammatory state. Independent effects acidosis on malnutrition: acidosis may lead to malnutrition in MHD patients with decreased appetite, increased protein catabolism and decreased synthesis. Independent effects of inflammatory states on malnutrition: inflammatory cytokines such as TNF-α and interleukin-6 are elevated in hemodialysis patients, and these factors exacerbate malnutrition by promoting proteolysis, inhibiting protein synthesis, and affecting energy metabolism. The interaction of acidosis and inflammation: acidosis may exacerbate the inflammatory response by affecting immune cell function and increasing oxidative stress, while inflammatory cytokines may also worsen acidosis by impairing renal function and promoting the production of acidic metabolic products. Inflammation affects the patient’s PEW status, so it is important to pay attention to the patient’s inflammatory status when correcting the patient’s nutritional status. Inflammation levels in patients with MHD may fluctuate over time, and changes in the frequency and quality of dialysis may affect inflammation levels. Regular monitoring and individualized treatment regimens are essential for effective management and control of inflammation.

### 3.4. Laboratory test indicators

Hsu^[27]^ recruited 647 MHD patients and this study revealed for the first time a negative correlation between serum chromium level and malnutrition in a population of MHD patients, which may be due to changes in gastrointestinal absorption, changes in appetite and thus changes in serum chromium level absorption. It has also been pointed out that decreased fat content, anemia, hypoproteinemia, and calcium and phosphorus metabolism disorders are also important influencing factors of PEW.^[28]^ Peng et al^[29]^ calculated the somatic cell mass index by somatic cell mass/height2 and classified all the study subjects into 4 groups (G1, G2, G3, and G4) based on the somatic cell mass index quartiles, and the risk of PEW development in the G1 group was 2.69 times higher than that of the patients in the G4 group.

### 3.5. Depression

The prevalence of depression in MHD patients has been increasing, reaching 22.8% to 62.0%.^[30]^ Johansson^[31]^ points out that less social activity and depression both affect energy and protein intake in dialysis patients. Markaki^[32]^ evaluated the depression and personality traits of 52 MHD patients through a questionnaire survey, depression and introverted personality were negatively correlated with the nutritional status of MHD patients, indicating that depression and personality traits were important factors influencing the nutritional status of MHD patients. Guenzani^[33]^ investigated and analyzed 132 MHD patients and found that the severity of depressive symptoms and cognitive impairment was related to the nutritional status of MHD patients. Mantzorou^[34]^ included 2092 patients with MHD from 7 cities, malnutrition was more common in patients with cognitive impairment and depressive symptoms, and cognitive status and depression were also independent influences on malnutrition in MHD patients. Therefore, there is a need to improve the poor emotional state of MHD patients, which in turn improves their nutritional status and reduces the incidence of PEW.

## 4. Nutritional intervention

### 4.1. Personalized nutritional counseling

The aim of dietary counseling is to guide patients to make the best choices to avoid overly prescriptive or restrictive diets and to provide practical advice that takes into account psychological and cultural needs and personal goals, and is monitored regularly to ensure that the patient’s needs are met. Prevention and management of malnutrition should focus on strategies to reduce symptom burden, optimize dietary intake, enhance dietary enjoyment and promote healthy bowel function as well as support physical function and independence.^[19]^ Portuguese scholars Garagarza^[35]^ conducted a multicenter 6-month longitudinal study that recruited 731 patients with MHD from 34 dialysis units and used personalized nutritional counseling (PNC) as an intervention, which showed significant reductions of the results showed that PNC significantly reduced the incidence of hyperkalemia, hyperphosphatemia, and improved Ca/P ratio and dialysis adequacy. To assess the effect of PNC on the nutritional status of MHD patients, Kim^[36]^ intervened on 42 MHD patients from 2 hospitals for 10 months and showed a significant increase in serum albumin levels. This study suggests that sequential PNC contributed to an improvement in protein intake, an improvement in serum albumin levels and a delay in muscle wasting, which had some positive impact on nutritional status.

### 4.2. Health education program

Develop and provide targeted and multifaceted health education programs and measures to help patients improve their nutritional status by making them aware of the risk of malnutrition and increasing relevant knowledge.^[37,38]^ Hernandez^[39]^ randomly assigned 120 ESRD patients receiving hemodialysis to the nutritional education program (NEP) group or oral supplementation (OS) group. Patients assigned to the NEP group received a 4-month education program designed to improve general nutritional knowledge, including cooking advice and well-designed menus, the OS group received nutritional supplements during hemodialysis, and after 4 months of intervention, all patients in the NEP group had increased nutritional knowledge (*P* < .05). Total protein serum values and other biochemical parameters were significantly improved in both groups. NEP is as effective as OS in preventing and improving malnutrition in MHD patients, and from a long-term perspective, NEP increases patients’ knowledge of relevant nutrition, reduces the financial burden of MHD patients, and saves medical resources.

### 4.3. Oral nutritional supplements during dialysis

Medical nutritional therapies also play an important role in preventing and treating PEW, electrolyte imbalances, and bone and mineral abnormalities.^[40]^ Oral nutritional supplements (ONS) can significantly reduce the readmission rate of MHD patients and have a favorable impact on the economic cost of health care.^[31]^ ISRNM emphasized in its 2017 consensus that for patients without contraindications, meals during hemodialysis should be considered part of standard of care practice.^[41]^ Benner^[42]^ conducted a retrospective analysis of ONS patients from 408 institutions, and the results showed that ONS played a positive role in reducing the mortality of MHD patients and improving certain nutritional status indicators, which supported the use of ONS during dialysis. Sohrabi^[43]^ used 220 mL vitamin E-fortified whey drink (15 g whey protein concentrate + 600 IU vitamin E), 220 mL whey drink (15 g whey protein concentrate), vitamin E and control group respectively during the dialysis process for MHD patients. The results show that the whey protein and vitamin E supplement of the new whey drink can improve the nutritional status of MHD patients in the short term. Tomayko^[44]^ supplemented whey or soy products for 6 months in dialysis for MHD patients, and the results showed that inflammation in MHD patients could be reduced and physical function improved. Ocepek^[45]^ pointed out that short-term ONS supplementation has a greater impact on the nutritional status of malnourished MHD patients, which may be because patients with poor nutritional status have a more urgent need for protein and energy intake to maintain the body’s energy requirements. Beddhu^[46]^ stated that among MHD patients with elevated inflammatory markers but otherwise mostly normal nutritional parameters, ONS was effective in increasing protein intake but had no effect on nutritional status or quality of life, thus inflammation in MHD patients also needs to be monitored with a focus in clinical work. Current research has demonstrated that ONS supplementation during dialysis improves the nutritional status of patients, but eating during dialysis may lead to alterations in the blood flow status of the patient, which in turn may lead to hypotension, and thus supplementation with ONS during dialysis may still require evaluation of the patient under multidisciplinary cooperation, which in turn will ensure the safety of the patient during the dialysis process.

### 4.4. Oral nutritional supplements combined with exercise during dialysis

Zang^[47]^ showed that exercise would not increase the burden on the kidney, but could also reduce the inflammatory state and improve the nutritional and clinical indicators of patients. Clarkson study^[48]^ found that both resistance exercise and aerobic exercise can improve objective indicators of physical function in MHD patients, as well as improve nutritional status and physical function in MHD patients, possibly because physical activity and exercise increase the rate of skeletal muscle protein synthesis. Martin^[49]^ found that ONS + resistance exercise group could better improve the nutritional status and physical function of MHD patients, followed by ONS + aerobic exercise group, and finally ONS. The 2020 KDOQI Nutrition Clinical Practice Guidelines for Chronic Kidney Disease point out that to maintain a stable nutritional status, the dietary protein intake of MHD patients is 1.0 to 1.2 g/kg per day. Consume 25 to 35 kcal/kg of energy per day. While protein and energy intake in most current MHD patients is usually lower than recommended by guidelines, ONS + aerobic is taken during dialysis exercise can not only improve the nutritional status and physical function status of patients, but also can be tried on the basis of ensuring patient safety.

### 4.5. Intradialytic parenteral nutrition

Intradialytic parenteral nutrition (IDPN) refers to the intravenous infusion of essential nutrients during MHD treatment.^[50]^ Marsen^[51]^ conducted a prospective, multicenter randomized trial on 107 MHD patients with PEW, and the results of this trial demonstrated for the first time that IDPN treatment resulted in a statistically significant and clinically meaningful increase in mean serum prealbumin, which was superior to PNC. However, it is emphasized that IDPN should only be used as a supplement in patients with spontaneous oral nutrient intake of at least 25 kcal/kg per day and protein intake of 0.9 g/kg. Anderson pointed out that IDPN did not improve relevant clinical outcomes in MHD patients compared to dietary counseling or oral supplementation.^[52]^ While the advantages of initiating an IDPN are that it is given during hemodialysis treatment, thereby reducing the risk of overload and avoiding additional burden on patients in terms of time investment or nutritional needs, the disadvantages of IDPN are increased risk of metabolic and electrolyte abnormalities, increased costs, and lack of data on improved clinical outcomes. There are also some practical difficulties in the implementation of IDPN in MHD patients. The use of IDPN is challenged not only by clinical issues but also by operational and administrative challenges specific to this setting. First, clinical aspects of IDPN setup: the addition of IDPN to hemodialysis requires careful planning and equipment setup by professionals specifically trained in hemodialysis administration. Secondly, cost is one of the major factors affecting the use of IDPN in clinical practice, with different insurance policies covering different costs in China, the cost of IDPN is often much higher than that associated with ONS, and comparative efficacy studies have shown that the 2 therapies have similar benefits.

## 5. Conclusion

MHD patients have a high risk of PEW, and PEW is an independent risk factor for death in MHD patients, while early diagnosis and correction of malnutrition can prevent further deterioration of the patient’s condition. There are fewer domestic studies on the PEW status of MHD patients, with the following major deficiencies: (1) The current status of analysis and intervention of factors affecting PEW in MHD patients is still insufficient. (2) Advice on malnutrition in MHD patients is mainly provided by doctors in blood purification centers, and there is a lack of nutritional management methods under multidisciplinary cooperation. (3) Whether to eat or not to eat in the course of dialysis is still controversial, which creates difficulties for the interdialytic period of eating to improve nutritional status. (4) Currently, the use of IDPN is still controversial, with limited data from studies on adverse events or cost-effectiveness of IDPN, which may be a reasonable treatment option for patients with poor nutritional status or those who are unable to receive the recommended treatments, but the widespread use of IDPN is still not supported by evidence.

Suggestions: (1) A large-sample, multicenter study be conducted to analyze the factors influencing PEW in MHD patients. (2) Nutritionists cooperate with doctors and nurses to conduct whole-process nutritional monitoring and file management for patients, and perform nutritional risk screening for MHD patients at least once every 6 months. (3) An appropriate health education approach be taken to build up the motivation of patients to maintain their dietary behaviors, and all efforts be made to eliminate MHD patients with dietary restrictions. (4) Establish a nutritional management process for patients, such as a stepwise nutritional laddering approach: for MHD patients with a healthy nutritional status to maintain a healthy diet, for patients with insufficient energy intake, ONS can be used, enteral nutrition should be applied as the next step, and IDPN should only be used as a supplemental nutrition, and if these interventions are ineffective, then try total parenteral nutrition to correct the malnutrition status of patients and improve the quality of survival. (5) Specific anti-inflammatory interventions or therapeutic approaches that may be beneficial for managing PEW in MHD patients: using high-flux dialyzers and hemodiafiltration modes, can more effectively remove medium and large molecular inflammatory mediators. Consuming a diet rich in omega-3 fatty acids, antioxidants, and fiber may help reduce inflammation. Short-term use of corticosteroids may help control acute inflammatory responses.

## Author contributions

**Methodology:** Jiquan Zhang, Peimin Zhuang.

**Software:** Peimin Zhuang, Xingying Xu.

**Supervision:** Fan Xu, Wei Qing.

**Writing – original draft:** Jiquan Zhang, Fan Xu, Wei Qing.

**Writing – review & editing:** Jiquan Zhang, Fan Xu, Wei Qing.
